# Applying real-time quantitative PCR to diagnosis of freemartin in Holstein cattle by quantifying *SRY* gene: a comparison experiment

**DOI:** 10.7717/peerj.4616

**Published:** 2018-04-27

**Authors:** Qinghua Qiu, Taoqi Shao, Yang He, Aziz-Ur-Rahman Muhammad, Binghai Cao, Huawei Su

**Affiliations:** 1 State Key Laboratory of Animal Nutrition, College of Animal Science and Technology, China Agricultural University, Beijing, China; 2 University of Agriculture Faisalabad, Institute of Animal and Dairy Sciences, Faisalabad, Pakistan

**Keywords:** Heterosexual twin female, Freemartin, H-Y antigen, qPCR, *SRY*

## Abstract

**Background:**

Freemartinism generally occurs in female offspring of dizygotic twins in a mixed-sex pregnancy. Most bovine heterosexual twin females are freemartins. However, about 10% of bovine heterosexual twin females are fertile. Farmers mostly cull bovine fertile heterosexual twin females due to the lack of a practical diagnostic approach. Culling of such animals results in economic and genetic-material losses both for dairy and beef industry.

**Methods:**

In this study, a comparative test, including qualitative detection of *SRY* gene by polymerase chain reaction (PCR), quantitative detection of relative content of *SRY* by real-time quantitative polymerase chain reaction (qPCR), and quantitative detection of H-Y antigen, was performed to establish the most accurate diagnosis for freemartin. Twelve Holstein heterosexual twin females were used in this study, while three normal Holstein bulls and three normal Holstein cows were used as a positive and negative control, respectively.

**Results:**

Polymerase chain reaction results revealed that *SRY* gene were absent in three heterosexual twin females and only two of them were verified as fertile in later age. The qPCR results showed that relative content of *SRY* was more than 14.2% in freemartins and below 0.41% in fertile heterosexual twin females. The H-Y antigen test showed no significant numerical difference between freemartin and fertile heterosexual twin female.

**Discussion:**

Our results show that relative content of *SRY* quantified by qPCR is a better detection method for diagnosis of freemartin in Holstein cattle as compare to qualitative detection of *SRY* gene by PCR or quantitative detection of H-Y antigen. To the authors’ knowledge, this is the first time we applied qPCR to diagnosing freemartin by quantifying *SRY* gene and got relative *SRY* content of each freemartin and fertile heterosexual twin female. We concluded that low-level of *SRY* would not influence fertility of bovine heterosexual twin female.

## Introduction

Freemartinism is an intersexual syndrome occurring in the female offspring of mixed-sex pregnancy, being one of the most important types of disorders of sexual development in domestic cattle (Padula, 2005; Villagómez et al., 2009). The most common consequences of freemartinism are masculinized phenotype and sterility (Jeon et al., 2012). Freemartin is a sterile female originated from heterosexual multiple pregnancies (Esteves, Båge & Payan-Carreira, 2012), usually born as co-twin to a male calf. Despite a rare event, freemartin cases have been reported born as triplets (Zobel, 2011), quadruplets (Biswas et al., 2015), amorphus globosus (Weber, Rudolph & Freick, 2017) and singleton which is due to loss of her male co-twin before parturition (Kitahara et al., 2002; Szczerbal et al., 2014).

The sterility of freemartin is explained through hormones and XX/XY chimera (Capel & Coveney, 2004; Freeman, 2007; Gonelladiaza et al., 2012). In the former scenario, male hormones such as testosterone and anti-Müllerian, originally due to Wolffian development and Müllerian regression (Loriaux, 2016), affect the female fetus through placental vascular anastomoses at 30–40 days of gestation and result in transformation of the general somatic habitus in female (Jiménez, Barrionuevo & Burgos, 2013; Ayalavaldovinos et al., 2014; Refaat, Ali & Tharwat, 2015). The chimera scenario explains that the XY cells of a male fetus transfer to his female twin through the shared vascular anastomosis branches, and result in formation of XX/XY chimeras which hinder the development of female gonads (Peretti et al., 2008).

It is observed that 82–97% of bovine females from heterosexual twins are freemartin and contain XX/XY chimera (Zhang et al., 1994; Fujishiro et al., 1995; Esteves, Båge & Payan-Carreira, 2012; Kozubska-Sobocińska, Danielakczech & Rejduch, 2016). The twinning rate is rising in last few years (Szczerbal et al., 2014) and it is expected that the number of fertile heterosexual twin female is also increasing. Normally heterosexual twin females are sold for meat or fetal bovine serum production which is economic and genetic material loss (Hirayama et al., 2007).

A rapid, sensitive, and inexpensive method for identification of freemartin at birth or early age becomes an urgent need to reduce unnecessary economic losses and to preserve important hereditary material, also to avoid the delivery of detrimental genetic materials by progeny (Biswas et al., 2015). Many diagnostic methods have been established for identification of freemartin, such as measurement of vaginal length (Khan & Foley, 1994), blood grouping test for degree of hemolysis (Kästli & Hall, 1978), karyotype analysis for XX/XY chimera (Dunn, Johnson & Quaas, 1981), polymerase chain reaction (PCR) or improved PCR techniques for detection of specific fragments located on the Y chromosome (Hirayama et al., 2007; Ron et al., 2010; Ayalavaldovinos et al., 2014), fluorescence in situ hybridization technique for detection of Y chromosome (Sohn et al., 2007; Villagómez & Pinton, 2008; Rubes et al., 2009), quantitative detection of hormones such as progesterone, estradiol, and anti-Müllerian hormone (Rota et al., 2002; Cabianca et al., 2007; Remnant et al., 2014; Kitahara et al., 2018), and detection of H-Y antigen qualitatively (Wachtel et al., 1980). Most of the methods are based on the assumption that freemartin contains XX/XY chimera and H-Y antigen, whereas fertile heterosexual twin female does not contain XX/XY chimera or H-Y antigen. However, studies reported that three XX/XY chimeric heterosexual twin females were fertile (Eldridge & Blazak, 1977; Smith, Camp & Basrur, 1977; Fujishiro et al., 1995). Fertile chimeras could be an accidental phenomenon but it motivated us to design a more accurate and efficient diagnostic approach to recognize fertile heterosexual twin female and to assist farmers with their decision making with respect to heifer selection.

The sex-determining region Y (*SRY*) gene is located in the sex-determining region of Y chromosome, and its sequence is highly conserved and easily adapted to quantitative assays (Nelson, 2002; Harley, Clarkson & Argentaro, 2003). H-Y antigen, another potential marker for identification, is a male tissue-specific antigen secreted by the testis (Wolf, 1998), and was previously used to determine sex of bovine embryos (Wachtel, 1979). The objective of the current study was to establish an effective diagnostic approach for freemartin by either qualitative and quantitative detection of the *SRY* gene or quantitative detection of H-Y antigen. We hypothesized that cell exchange also occurred in fertile heterosexual twin female at low levels, thus slight cell chimera would not influence reproductive capacity of heterosexual twin female.

## Materials and Methods

### Ethic statements

All animals used in this study were handled strictly in accordance with the recommendations in the Guide for the Care and Use of Laboratory Animals of the National Institutes of Health of China. The protocols were approved by the Animal Welfare Committee of China Agricultural University (Permit Number: DK1008).

### Animals and samples

A total of 18 Holstein cattle of age 20 months ± 10 days (12 Holstein heterosexual twin females, three normal Holstein bulls and three normal Holstein cows) were selected in this study. All animals were on the same feeding regime and at same colony house. Animals were reared for two years. Blood samples were collected into sterile tubes with anticoagulant of EDTA by venipuncture for molecular biological analysis. For H-Y antigen determination, blood samples were collected into vacuum tubes without anticoagulant, and centrifuged at 3,500 rpm for 15 min before pipetting the supernatant serum into a 1.5 mL centrifuge tube. All blood samples and serum samples were transported back to the laboratory on ice within 6 h for subsequent DNA extraction and H-Y antigen determination, respectively.

### Genomic DNA isolation and primer design

All blood samples were kept on a clean bench at room temperature for 10 min before being isolated for DNA according to the manufacturer’s instructions (Tiangen Biotech, Beijing, China). The DNA concentration of all samples was determined by NanoDrop 2000 (ThermoFisher Scientific, Waltham, MA, USA) and then diluted to 50 ng/μL with buffer TB (Tiangen Biotech, Beijing, China). Primers (Table 1) of the target gene (*SRY)* and housekeeping gene (*GAPDH*) for PCR and real-time quantitative polymerase chain reaction (qPCR**)** assays were designed by Primer 5.0 and were synthesized by Sangon Biotech (Shanghai, China).

**Table 1:** Primer sequences for PCR and real-time quantitative PCR.

Gene	Sequence (5′ to 3′)	Amplicon size (bp)	Tm^1^ (°C)	Accession No.
*SRY*	Forward: GCCACAGAAATCGCTTCC	229	60	NC_016145.1
	Reverse: CCGTGTAGCCAATGTTACCTT			
*GAPDH*	Forward: GTGAGAGACGGAACAGGAAGAA	110	60	AC_000162.1
	Reverse: ATGAGGGAAGACAGGACAAAGC			

**Note:**

1 Melting temperature.

### PCR and relative quantitative PCR

Well optimized 20 μL reaction system was as follows: 10 μL of 2 × PCR FastStart Universal SYBR Green Master (ROX) (Roche, Mannheim, Germany), 0.3 μL of each primer (10 μM), 1 μL of DNA template and 8.4 μL of DNase/RNase-Free water (Tiangen Biotech, Beijing, China). The PCR conditions included an initial incubation at 94 °C for 3 min, followed by 30 cycles of 94 °C for 15 s, 60 °C for 30 s, 72 °C for 15 s, and the final step of 72 °C for 5 min, amplified products were sent to Sangon Biotech (Shanghai, China) for Sanger sequencing. The qPCR program, executed using a Comparative Quantitation (Calibrator) Real-time PCR System, included a 10 min polymerase activation step at 95 °C followed by a 3-step PCR, which consisted of 40 cycles (95 °C × 15 s, 60 °C × 60 s, 72 °C × 15 s), and a melting curve was generated for the specificity assessment of each pair of primers. Both PCR and qPCR assays were performed in the Stratagene Mx3000P (Agilent Technologies, Wilmington, DE, USA), and qPCR was done in triplicate. Relative content of *SRY* was calculated using the comparative CT (2^−ΔΔCT^) method (Schmittgen & Livak, 2008) with normal bull as calibrator and *GAPDH* as the internal control to normalize the data, where CT refers to cycle threshold. ΔCT was calculated by subtracting the CT values of *GAPDH* from the CT values of the *SRY* of interest. ΔΔCT was then calculated by subtracting mean ΔCT of the normal bull from ΔCT of tested heterosexual twin female, and relative content of *SRY* was calculated by the equation 2^−ΔΔCT^.

### H-Y antigen quantification

H-Y antigen concentration was determined by Bovine H-Y Ag ELISA Kit (MLBio, Shanghai, China). Competition assay in enzyme-linked immunosorbent assay (ELISA) was adopted in the determination. All samples were evaluated in a 96-well microtiter plate in duplicates, and the mean value of each sample was calculated. All steps were conducted by the same laboratory technician according to the manufacturer’s instructions. Absorbance values were obtained by using an ELISA reader (Tecan, Männedorf, Zürich, Switzerland) at a wavelength of 450 nm.

### Statistical analysis

H-Y antigen concentration was calculated by Dose–Response Regression Models of Curve Expert Professional Version 2.6.3 Program (Hyams D.G., Starkville, MS, USA). Data were considered as valid only when *R*^2^ > 0.99 in the standard curve.

## Results

**Figure 1:**
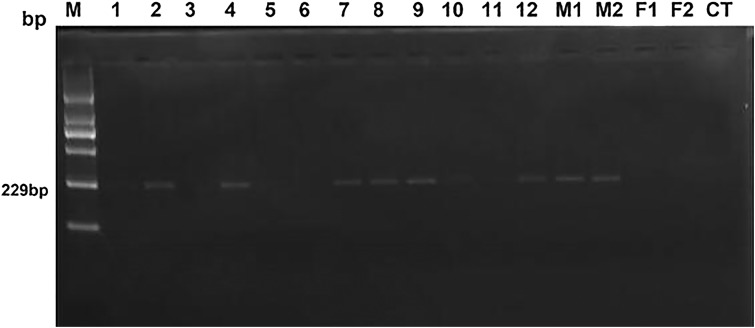
The electrophoresis results of test and control samples by PCR. Lane 1 (M): Molecular weight marker (2,000-bp DNA ladder); bands from bottom to top are listed as 100, 250, 500, 750, 1,000 and 2,000 bp; the size of target bands is 229 bp. Lanes 2 through 13 (No.1–No.12) display samples of 12 female heterosexual twins. Lanes 14 (M1) and 15 (M2) represent positive control. Lanes 16 (F1) and 17 (F2) show negative control. The rightmost lane (CT) is the blank control.

After a two-year breeding of heterosexual twin females with three normal bulls, only two heterosexual twin females were successfully pregnant. Both pregnant heterosexual twin females gave birth single male calves. Gel electrophoresis results and sequencing results in PCR, fertility and relative content of *SRY* are shown in Fig. 1 and Table 2. Based on Fig. 1, samples No. 3, No. 5, and No. 6, negative samples (F1 & F2) and blank control (CT) were evaluated as negative clearly, while samples No. 1 and No. 11 could not be judged as negative or positive with naked eye, others were obviously positive. However, only No. 3 and No. 6 were verified as fertile heterosexual twin females, whereas No. 5 was verified as sterile after natural mating for several times even though it was shown to be negative in PCR (Fig. 1).

**Table 2:** Detection results of amplifying *SRY* gene by PCR or qPCR and H-Y antigen determination.

Sample No.	*SRY* in electrophoresis^1^	Sequencing alignment^2^	Relative content of *SRY*^3^(%)	H-Y antigen (pg/mL)	Fertility^4^
1	Unknown	Consistent	20.7	1.89	N
2	Positive	Consistent	61.4	1.26	N
3	Negative	No product	0.41	1.03	Y
4	Positive	Consistent	41.4	1.04	N
5	Negative	Consistent	18.5	1.17	N
6	Negative	No product	0.09	1.00	Y
7	Positive	Consistent	59.0	1.28	N
8	Positive	Consistent	61.5	1.06	N
9	Positive	Consistent	53.6	1.04	N
10	Positive	Consistent	14.2	1.52	N
11	Unknown	Consistent	25.0	1.01	N
12	Positive	Consistent	88.9	1.69	N
M^5^	Positive	Consistent	100	37.1	Y
F^6^	Negative	No product	0	1.00	Y

**Notes:**

1 Result of positive and negative means the band of sample could be judged precisely as presence and absence, respectively. While result of unknown means the band of sample could not be judged precisely as negative or positive with the naked eye.

2 Sequencing alignment result of consistent means amplification sequence of sample is consistent with target sequence of *SRY*, and no product means no synthesis is detected in Sanger sequencing.

3 Relative content of *SRY* was calculated in accordance to the standard of normal bull as 100%.

4 Fertility was verified by calving or not after natural mating for several times over two years. Y represents fertile and N represents sterile.

5 M means bull with normal fertility, serving as the positive control, and content of H-Y antigen was the mean of three normal Holstein bulls.

6 F means cow with normal fertility, serving as the negative control, and content of H-Y antigen was the mean of three normal Holstein cows.

On the other hand, qPCR results showed that the relative content of *SRY* varied from 14.2% to 88.9% in freemartins, whereas 0.09% and 0.41% in fertile heterosexual twin females (Table 2).

Concentration of H-Y antigen was showed in Table 2. Average concentration of H-Y antigen in freemartin was 1.30 pg/mL, with a maximum of 1.89 pg/mL and minimum of 1.01 pg/mL, while 1.00 and 1.03 pg/mL in fertile heterosexual twin females, respectively.

## Discussion

All animals were on same feeding regime for two years. Environment and housing condition were also same throughout the experimental period. All heterosexual twin females had free access to mating after estrus. Therefore, it is excludable that subfertile female that requires more mating than female with normal reproduction would appear in our experiment. The obvious differences of qPCR results between freemartin and fertile heterosexual twin female indicate that quantifying *SRY* gene by qPCR technique might be a more accurate method for diagnosing freemartin. However, more cases are still needed to confirm the exact value of relative content of *SRY* between freemartin and fertile heterosexual twin female.

The frequency of XY cells in freemartin ranged from 2% to 99% in cytogenetic analysis (Peretti et al., 2008). However, improper diagnosis could occur if the frequency of XY cells in the heterosexual twin female is less than 5% in cytogenetic analysis (Eldridge & Blazak, 1977), and much below 1 in 500 by PCR-based assays (Mcniel et al., 2006). In our study, the relative *SRY* content of freemartin varied from 14.2% to 88.9%. This made the criterion ambiguous because congruent relationship between frequency of XY cells and relative content of *SRY* remained unknown. Previous reports have shown the relative proportion of XY to XX in karyotypes of freemartins was not related with the degree of masculinization (Peretti et al., 2008; Kozubska-Sobocinska et al., 2011; Szczerbal et al., 2014). It has also been reported that the proportion of XY cells had no relation to the inhibition degree of ovary or of Müllerian duct (Vigier, Prepin & Jost, 1972). However, no report has shown the relationships between fertility and degree of masculinization in heterosexual twin female, thus the relationship between XY cells and fertility of heterosexual twin female remains unknown. Our data showed the presence of XX/XY chimera in fertile heterosexual twin female, indicating low-level of *SRY* would not influence fertility of bovine heterosexual twin female. Moreover, previous researchers have proven that XY to XX karyotype ratio was stable afterbirth for freemartin (Greene, Dunn & Foote, 1977; Pessamorikawa, Niku & Iivanainen, 2004). Therefore, our results could be applied equally to the newborn calf.

Low levels of *SRY* represents the low possibility of gonadal dysplasia. Primordial-gonad cells of fetus are in a bipotential status after conception (Ottolenghi et al., 2007; Norling et al., 2013; Piprek et al., 2017), which means the possibility to differentiate to either male cells or female cells. Testis-determining factor encoded by the *SRY* gene initiates testis differentiation of male, mainly via upregulating *SOX9* and *FGF9* (Hiramatsu et al., 2009; Moniot et al., 2009). The bipotential-gonad cells begin to differentiate into sertoli cells and leydig cells once *SOX9* reached proper levels (Shoemaker et al., 2007; Benko et al., 2011), resulting in formation of the testis (Harikae et al., 2012). In that situation, low-level of *SRY* is inadequate to initiate testis differentiation because of insufficient *SOX9*. Therefore, bipotential-gonad cells would not differentiate into sertoli cells and leydig cells which are vital for males’ cell differentiation and proliferation (Rebourcet et al., 2014; Ryan, 2014). Anastomoses of two opposite-sex fetuses occur after the critical period of reproductive organ differentiation may lead to low-level of *SRY* (Szczerbal et al., 2014).

Interestingly, we observed certain H-Y antigen in all heterosexual twin females without any significant numerical difference between freemartin and fertile heterosexual twin female (Table 2). H-Y antigen was previously considered as male-specific cell-surface proteins (Muller, 1996). One possible explanation for the existing results is that H-Y antigen of the male fetus delivers to his co-twin female fetus via blood cells during the fetus period. The lack of numerical differences between freemartin and fertile heterosexual twin female indicated that quantitative detection of H-Y antigen was not suitable for selecting fertile heterosexual twin female. Our findings are in contradict with previous reported studies that a higher proportion of Y/X expressed more H-Y antigen (Wachtel et al., 1975; Fraccaro et al., 1982). Theoretically, concentration of H-Y antigen presents descending trend in normal bull, freemartin, and fertile heterosexual twin female. However, we only found that concentration of H-Y antigen in normal bull was higher numerically than freemartin and fertile heterosexual twin female, no visible difference between freemartin and fertile heterosexual twin female. Current results indicate that slight H-Y antigen would not hamper reproduction of a heterosexual twin female. The presence of H-Y antigen in fertile heterosexual twin female could be due to transfer of H-Y antigen to female fetus when hematopoietic tissue and cell interchange after vascular anastomoses during fetus period (Niku et al., 2007; Abuelo, 2009; Kozubska-Sobocińska, Danielakczech & Rejduch, 2016). It has been reported that H-Y antigen is activated during the formation of testes rather than triggering the formation (Wolf, 1998). Therefore, H-Y antigen not plays a decisive role in development of testis, and its presence not always leads to masculinization in female. Wachtel et al. (1976) found that H-Y antigen also existed in women with XX/XY chimera as well as in XX true hermaphrodites, and reported that H-Y antigen expressing in the blood not hamper the presence of ovaries in women with XX/XY chimera or hermaphrodite (Wachtel et al., 1976; Wachtel, 1977). It can be hypothesized that concentration of H-Y antigen in fertile heterosexual twin female may be nearly the same or slightly lower than that of freemartin.

An alternative assumption that cannot be ruled out is that discrete sample size hinders the difference of H-Y antigen between freemartin and fertile heterosexual twin female. More heterosexual twin females are needed to eliminate individual variability.

To the best of the authors’ knowledge, except for the qualitative experiment reporting in 2012 applying qPCR to quantifying *BRY4* gene to distinguish freemartin from fertile heterosexual twin female via characteristic fluorescence curve (Artigas et al., 2012), this is the first time we applied qPCR to diagnosing freemartin by quantifying *SRY* gene. Through this method, we got the relative *SRY* content of each freemartin and fertile heterosexual twin female. We declare that low-levels of *SRY* would not influence fertility of bovine heterosexual twin female.

## Conclusion

In summary, we concluded that quantifying *SRY* gene by qPCR is a better detection method for the diagnosis of bovine freemartin compared with PCR or quantitative detection of H-Y antigen. Freemartin contains high relative content of *SRY*, whereas a fertile heterosexual twin female contains slight relative content of *SRY*. Our results indicate that low-levels of *SRY* would not influence fertility of a bovine heterosexual twin female. Further research is needed to confirm a potential cutoff value of relative content of *SRY* at different growth stages before puberty to make a strategic decision for a heterosexual twin female.

## Supplemental Information

10.7717/peerj.4616/supp-1 Supplemental Information 1 Raw data of PCR, qPCR detection and H-Y antigen determination. Data include sequencing of sample, figure 1, H-Y antigen determination absorbance values, and relative content of SRY.

## Additional Information and Declarations

### Data Availability

The following information was supplied regarding data availability: The raw data are provided in the Supplemental File.
